# Morphine-mediated release of astrocyte-derived extracellular vesicle miR-23a induces loss of pericyte coverage at the blood-brain barrier: Implications for neuroinflammation

**DOI:** 10.3389/fcell.2022.984375

**Published:** 2022-11-21

**Authors:** Ke Liao, Fang Niu, Guoku Hu, Shilpa Buch

**Affiliations:** ^1^ Department of Pharmacology and Experimental Neuroscience, University of Nebraska Medical Center, Omaha, NE, United States; ^2^ Smidt Heart Institute, Cedars-Sinai Medical Center, Los Angeles, CA, United States

**Keywords:** drug abuse, miRNA, extracellular vesicles, pericyte, monocyte, neuroinflammation

## Abstract

Opioids such as morphine are the most potent and efficacious drugs currently available for pain management. Paradoxically, opioids have also been implicated in inducing neuroinflammation and associated neurocognitive decline. Pericytes, a critical component of the neurovascular unit (NVU), are centrally positioned between endothelial cells and astrocytes, maintaining function of the blood-brain barrier (BBB) nd regulating neuroinflammation by controlling monocyte influx under various pathological conditions. The role of pericytes in morphine-mediated neuroinflammation however, has received less attention, especially in the context of how pericytes crosstalk with other central nervous system (CNS) cells. The current study was undertaken to examine the effect of miRNAs released from morphine-stimulated human primary astrocyte-derived extracellular vesicles (morphine-ADEVs) in mediating pericyte loss at the blood-brain barrier, leading, in turn, to increased influx of peripheral monocytes. Our findings suggest that the heterogeneous nuclear ribonucleoprotein complex A2/B1 (hnRNP A2/B1) plays role in morphine-mediated upregulation and release of miR-23a in ADEVs, and through action of morphine *via* mu opioid receptor.We further demonstrated that miR-23a in morphine-ADEVs could be taken up by pericytes, resulting in downregulation of PTEN expression, ultimately leading to increased pericyte migration. Furthermore, both overexpression of PTEN and blocking the miR-23a target site at PTEN 3UTR (by transfecting miR-23a-PTEN target protector), attenuated morphine-ADEV-mediated pericyte migration. We also demonstrated that in the microvessels isolated from morphine-administered mice, there were fewer PDGFβR + pericytes co-localizing with CD31^+^ brain endothelial cells compared with those from saline mice. In line with these findings, we also observed increased loss of pericytes and a concomitantly increased influx of monocytes in the brains of morphine-administered pericyte-labeled NG2-DsRed mice compared with saline mice. In conclusion, our findings indicate morphine-ADEVs mediated loss of pericyte coverage at the brain endothelium, thereby increasing the influx of peripheral monocytes in the central nervous system, leading to neuroinflammation.

## Introduction

Breach of the blood-brain barrier (BBB) with increased influx of monocytes is a prominent hallmark feature of neuroinflammation (Floris et al., 2004; Bethel-Brown et al., 2012; Smith et al., 2018). A subset of circulating white blood cells—monocytes—can migrate across the BBB under pathological conditions into the CNS. This has been implicated as a source of neuroinflammation and progression of many central nervous system (CNS) neurodegenerative diseases, such as Parkinson’s disease (PD) (Wijeyekoon et al., 2018), Alzheimer’s disease (AD) (Theriault et al., 2015), multiple sclerosis (MS) (Filion et al., 2003), and HIV-associated neurocognitive disorders (HAND) (Yao et al., 2010; Burdo et al., 2013; Napuri et al., 2013; Yadav et al., 2016; Veenstra et al., 2017). Along these lines, morphine has been shown to not only enhance HIV-1 infectivity of monocyte-derived dendritic cells and macrophages (Reynolds et al., 2012), but can also facilitate monocyte infiltration into the brain, leading, in turn, to the enhanced progression of HIV-associated neuropathology in morphine-dependent, SIV-infected rhesus macaques (Bokhari et al., 2011; Dutta and Roy, 2015). The underlying mechanisms by which morphine elicits these responses, however, remain poorly understood.

Several studies have demonstrated that morphine induces increased release of proinflammatory mediators in brain vascular endothelial cells and can also lead to breach of the endothelial barrier (Mahajan et al., 2008; Wen et al., 2011; Wang et al., 2012). Pericytes are uniquely positioned cells within the neurovascular unit (NVU) that play vital roles in the development and maintenance of the BBB (Sweeney et al., 2016). Pericytes are emerging as essential integrators, coordinators, and effectors of the BBB, wherein they regulate the permeability of the barrier, the cerebral blood flow, and toxic byproduct clearance (Niu et al., 2014). Recent studies demonstrated that pericyte loss in neurological disorders results in increased extravasation of peripheral immune cells as well as elevated levels of sPDGFRβ (soluble Platelet-derived Growth Factor Receptor-β) in the cerebrospinal fluid (CSF) (Nation et al., 2019). These findings underscore a novel role of pericytes in neurocognitive disease and indicate pericyte loss as a biomarker of human cognitive dysfunction (Montagne et al., 2015; Nation et al., 2019).

Extracellular vesicles (EVs), comprising of microvesicles (50 nm–1 μm), exosomes (30–150 nm) and apoptotic bodies (50 nm–5 μm), are lipid bilayer-delimiting particles secreted from diverse cell types that facilitate intercellular communication among different cell types and tissues (Hu et al., 2016; Lee et al., 2018). EVs are internalized by recipient cells following receptor-ligand interactions and regulate the signaling pathways by transporting their cargo, such as proteins, lipids, and RNAs to recipient cells (Abels and Breakefield, 2016). MiRNAs are small and evolutionarily conserved noncoding RNAs and appear as important post-transcriptional regulators of gene expression in mammalian cells (Felekkis et al., 2010). Numerous studies aimed at examining the role and function of EV-miRNAs in various diseases, including cardiovascular disease (Zhu et al., 2016), cancer (Desmond et al., 2019; Moloney et al., 2020), chronic lung disease (Go et al., 2020), as well as neurocognitive diseases (Shah et al., 2017; Yang et al., 2018; Hu et al., 2020), have identified critical EV-miRNAs as novel biomarkers of these diseases. Moreover, the dysregulation of several miRNAs has been associated with viral-induced neuroinflammation and neurodegenerative processes (Mishra et al., 2012). In our previous study, we found 15 out of 1,079 miRNAs that significantly upregulated in morphine-ADEVs, that were linked to inhibition of morphine-mediated microglial phagocytosis in recipient cells *via* activation of toll-like receptor 7/8 (TLR7/8) (Hu et al., 2018). More recently, ADEV-miRNAs-mediated regulation of other CNS cells such as neurons (Hu et al., 2020), microglia (Chaudhuri et al., 2018; Liao et al., 2020; Panaro et al., 2020), endothelial cells (Dickens et al., 2017) has been gaining momentum. The crosstalk between astrocytes and pericytes however, has not been explored in depth.The mechanisms by which ADEV-miRNAs regulate the function of pericytes remains especially poorly understood. The current study was aimed at examining the yet undefined role of miRNA cargo in morphine-ADEVs in regulating pericyte function(s) under the condition of morphine-mediated neuroinflammation.

In the present study, we demonstrated upregulation of miR-23a in morphine-ADEVs, which, upon uptake by pericytes, resulted in activation of the PTEN/Akt pathway, ultimately culminating into pericyte migration and its loss from the endothelium. These findings thus underscore a therapeutic strategy for future treatment of morphine-mediated neuroinflammation.

## Materials and methods

### Reagents

Morphine was purchased from R&D Systems (Minneapolis, MN, United States). Chemical inhibitors, including the opioid receptor antagonist naltrexone, mimic and inhibitor of hsa-miR-23a-3p were purchased from Sigma-Aldrich (St. Louis, MO, United States). The hnRNP A2/B1 siRNA (h): sc-43841 were purchased from Santa Cruz. Cell Tracker Green CMFDA and Red CMTPX were purchased from Invitrogen.

### Animals

C57BL/6N WT mice (male, 6–8 weeks) were purchased from Charles River Laboratories, Inc. (Wilmington, MA). Mice with neural/glial antigen-2 expression (NG2 DsRed) provided red fluorescent-labeled pericytes for detecting *in vivo*. DesRed-NG2 mice were purchased from Jackson Labs (Bar Harbor, ME) from stock Tg (Cspg4-DsRed.T1)1Akik/J. As described by the provided company: NG2DsRedBAC transgenic mice express DsRed.T1 (a red fluorescent protein variant) under the control of the mouse NG2 (Cspg4) promoter/enhancer. Archival brain tissues from morphine-administered or non-administered rhesus macaques were used for brain microvessels isolation in this study. Macaques were exposed to morphine as described previously (Sil et al., 2018).The macaques (N = 4) were gradually acclimated to morphine by starting with an initial dose of 6 mg/kg body weight for 1 week and escalating to a final dose of 12 mg/kg (in 3 mg/kg increments per week) for the remaining 10 weeks. For the control group, macaques (N = 4) received saline for 12 weeks. All the animals were housed under constant temperature and humidity conditions on a 12 h light, 12 h dark cycle, with lights on at 07:00. All animal procedures were performed following the protocols approved by the Institutional Animal Care and Use Committee at the University of Nebraska Medical Center.

### Cell cultures

Human primary astrocytes were purchased from ScienCell Research Laboratories (Carlsbad, CA, United States), and the cells were cultured in astrocyte medium (ScienCell). For our study, we used the cells within 10 passages.

Primary human brain vascular pericytes (HBVPs) were purchased from ScienCell and cultured in the pericyte culture medium (ScienCell). HBVPs were isolated from human fetal brain. The purity of pericyte was validated (>98% purification) as reported in our previous study (Niu et al., 2019), by positivity for PDGFR-β, nerve/glial antigen 2 (NG2), Desmin, and T-Box Transcription Factor 18 (TBX18) that are specific pericyte markers. Additionally, pericytes also showed negligible staining for smooth muscle alpha-actin (αSMA) as well as the endothelial cell marker CD31. Cells were cultured in dishes coated with poly-l-lysine (2µg/cm2; ScienCell), and cells were used within passages 2–5.

### EV isolation

We isolated EVs from the culture media of primary astrocytes using differential centrifugations as previously described (Ye et al., 2018). In brief, culture media were collected, centrifuged at 1,000 × g for 15 min to get rid of live cells, and again spun at 10,000 × g for 30 min, followed by filtration of the supernatant through a 0.22-µm filter to get rid of cell debris. EVs were pelleted by ultracentrifugation (Beckman 32Ti rotor; Beckman Coulter, Brea, CA, United States) at 100,000 × g for 70 min. We characterized the EVs using a BCA Protein Assay Kit (Pierce, Rockford, IL, United States) to check protein content, WB to check exosome markers such as Alix, TSG101, and CD63. EVs were further quantified using ZetaView Particle Metrix, as previously reported (Liao et al., 2020).

### Oligos and plasmid transfection

Under The RNA, oligos (miR-23a sequence: AUC​ACA​UUG​CCA​GGG​AUU​UCC) were purchased from Integrated Technologies (Coralville, Iowa). The custom-designed miR-23a-PTEN-Target Protector negative control (miR-23a-TP-ctrl: scramble sequence: 5′ GCC​ATC​AAA​CTC​TAT​AAA​TGC​TGC​TCT 3′) and miR-23a-PTEN-Target Protector (miR-23a-PTEN-TP: 5′ CTT​CAC​ATT​AGC​TTT​ACA​ATA​GTA​GTT 3′) were purchased from Integrated DNA Technologies. EVs were loaded with oligos using Exo-Fect Exosome Transfection Reagent according to the manufacturer’s instructions. Anti-miR-23a were obtained from Integrated Technologies. pEF6. mCherry-TSG101 (Addgene plasmid 38318) was a gift from Dr. Quan Lu (Harvard School of Public Health, Boston, MA).

### Luciferase activity assays

As described in our previous study (Yang et al., 2018), we did the modification. Briefly, a 38 bp PTEN 3′UTR segment (sense 5’ - tcg​agg​cgg​ccg​cCT​ACT​ATT​GTA​AAG​CTA​ATG​TGA​AT-3′ and antisense 5′- CTA​GAT​TCA​CAT​TAG​CTT​TAC​AAT​AGT​AGG​CGG​CCG​CC-3′) containing the putative miR-23a target site was cloned into the XhoI and XbaI sites of the pmirGLO vector. For pmirGLO-PTEN 3′UTR-miR-23a-target-mutant segment (sense 5′- tcg​agg​cgg​ccg​cCT​ACT​ATT​GTA​AAG​CTT​TAC​ACT​AT-3′ and antisense 5′- CTA​GAT​TCA​CAT​TAG​CTT​TAC​AAT​AGT​AGG​CGG​CCG​CC-3′), the miR-23a target site (AATGTGA) within the PTEN 3′UTR was changed to (TTACACT). Followed the manufacturer’s protocol (Promega), seeding the HEK293 cells into 24-well plates. Co-transfect either miR-23a mimic or scramble miRNA-control with either pmirGLO-PTEN-3′UTR-miR-23a-target or pmirGLO-PTEN-3′UTR-miR-23a-target-mutant luciferase reporter vector using lipofectamine 3000 (Invitrogen) for 2 days, followed by an assessment of the luciferase activity using the Dual-Luciferase Reporter Assay (Promega). We use renilla luciferase activity to normalize firefly luciferase activity (three independent experiments, performed in 3 wells each time).

### Western blotting

Brain tissues and treated cells were lysed using the Mammalian Cell Lysis kit (Sigma-Aldrich), as described previously (Liao et al., 2020). Proteins in equal amounts were electrophoresed in an SDS-polyacrylamide gel under reducing conditions followed by transfer to PVDF membranes. Blots were blocked with 3% BSA in TBS-Tween for 1h, followed by an incubation of antibodies specific hnRNP A2/B1 (1:1000; sc-53531; Santa Cruz), PTEN (1:1000; ab32199; Abcam), p-Akt (1:1000; 9271S; Cell Signaling), Akt (1:1000; 9272S; Cell Signaling), GAPDH (1: 5000, #5174; Cell Signaling) and β-actin (1:5,000; A5316; Sigma-Aldrich). Secondary antibodies were alkaline phosphatase-conjugated to goat anti-mouse/rabbit IgG (1:10,000; Jackson ImmunoResearch Labs). Signals were detected by SuperSignal West Dura Extended Duration or Pico PLUS Chemiluminescent Substrate (Thermo Fisher Scientific). All experiments had at least four biological replicates, and representative blots are presented in the figures.

### Real-time PCR

Comparative real-time PCR was performed with the use of Taqman universal PCR Master Mix (Applied Biosystems) for quantitative analysis of mRNA expression. Specific primers and probes for mature miR-23a and pri-miR-23a and snRNA RNU6B (U6) were obtained from Applied Biosystems. All reactions were run in triplicate. The amount of miRNA was obtained by normalizing to snRNA RNU6B relative to control as previously reported (Hu et al., 2017).

### *In situ* hybridization and immunostaining

Human primary astrocytes were fixed and prehybridized in hybridization buffer (50% formamide, 200 μg ml−1 yeast tRNA, 10 mM Tris-HCl, pH 8.0, 1 × Denhardt’s solution, 600 mM NaCl, 1 mM EDTA, 0.25% SDS, 10% Dextran sulfate) at a concentration of 9 p.m. for the commercially available digoxigenin-labeled miR-23a probe (Exiqon). LNA-modified miR-23a, labeled at both the 5′ and 3′ ends with digoxigenin (Exiqon), followed by dilution to the final concentration at 2 p.m. in hybridization buffer. Subsequently, the probes in hybridization buffer were added and incubated in a humid chamber overnight at 37°C. Then, we washed the slides three times in 2 × SSC for 2 min each at 42°C and in 0.2 × SSC for 2 min each at 42°C. The slides were then blocked with blocking buffer (3% normal goat serum and 1% bovine serum albumin in 1 × PBS) for 1 h at room temperature, followed by incubation with anti-digoxigenin conjugated with horseradish peroxidase (1:100, Roche Diagnostics, Mannheim, Germany) and anti-GFAP (1:200, G3893, Sigma-Aldrich) antibodies overnight at 4°C. The slides were washed twice with PBS, followed by incubation with Alexa Fluor 488 goat anti-rabbit IgG (1:200, Invitrogen, Carlsbad, CA) antibody for 1 h at room temperature. Then washed twice in PBS and the signal amplification using TSA Cy5 kit (PerkinElmer, Waltham, MA). Then, we mounted the slides using Prolong gold anti-fade reagent with DAPI (Invitrogen).

### Immunostaining and image analysis

The samples on the slides were fixed with 4% formaldehyde for 20 min at room temperature. We gave the slides three times wash with PBS, followed by permeabilization with 0.3% Triton X-100 for 30 min, rewashing three times, and blocking in 10% goat serum in PBS for 2 h at room temperature. The following antibodies were used for immunostaining: PDGFR-β (1:1000; ab32570; Abcam) and CD31 (1:1000; ab24590; Abcam). The slides were washed three times with PBS, followed by incubation with Alexa Fluor 488–conjugated anti-rabbit or anti-mouse (Invitrogen) for 1 h at room temperature. After a final washing with PBS three times, the slides or coverslips were mounted using Prolong Gold Antifade Reagent (Invitrogen). Fluorescent images were taken at room temperature on a Zeiss Observer, under the condition of a Z1 inverted microscope with a 40 × /1.3 or 63 × /1.4 oil-immersion objective. The images were analyzed with ImageJ software.

### Brain microvessel isolation

Mouse and macaque brain microvessels were isolated following a differential centrifugation protocol as described previously (Niu et al., 2015). Briefly, the brains were removed and immediately immersed in ice-cold isolation buffer A (4.7 mM KCl, 103 mM NaCl, 2.5 mM CaCl2, 1.2 mM MgSO4, 1.2 mM KH2PO4, and 15 mM HEPES, pH 7.4), followed by the removal of the cerebellum, olfactory bulb, and meninges. Subsequently, the brains were homogenized in 2.5 ml of isolation buffer B (10 mM glucose, 25 mM NaHCO3, 1 mM Na pyruvate, and 10 g/L Dextran, pH 7.4) containing protease inhibitors. The homogenates were added to Dextran (6 ml; 26%) and centrifugated for 20 min at 5800 × g. Pellets were resuspended in isolation buffer B, followed by filtration through a 70-μm mesh filter. Isolated brain microvessels were then harvested from the filtered homogenates by centrifugation. Some pure brain microvessels were used for immuno-staining by spreading on glass slides and prepared for PECAM1/CD31, and PDGFR-β detection.

### Scratch test

According to the manufacturer’s instructions and our previous study (Niu et al., 2014), 600 μl cell suspension of HBVP (concertation: 4 × 10^5 cells/ml) was added to the 24-well with insert. After 24 h, the insert was removed to form a consistent scratch gap (0.9 mm). The cells were then treated with either control-ADEVs or Morphine-ADEVs for 16 h followed by fixation and staining. The effect of the Morphine-ADEV on gap closure was determined according to HBVP migration in the gap zone by microscope photographs using Olympus DP71 microscope. Statistical analysis on the percentage of gap closure was obtained using ImageJ.

### Bone marrow-derived monocyte isolation

According to our previous study (Niu et al., 2019), we used C57BL/6N mice (Jackson Laboratory), 6–8 weeks of age, as BMM donors. Briefly, first removed the femur, followed by dissociating bone marrow cells into single-cell suspensions. Sequentially, the single-cell suspensions of bone marrow cells were cultured for 5 days supplemented with 1,000 U/ml of macrophage colony-stimulating factor.

### Trans-well migration assay

#### Endothelial cell-pericyte 3D coculture model

Loss of pericyte coverage on endothelial cells was determined using an *in vitro* endothelial cell-pericyte 3D coculture model. Briefly, BD Matrigel matrix (BDbioscience cat No. 354234) was thawed overnight on the ice at 4 °C . The thawed Matrigel™ solution was kept in an ice bath and transferred (80 μl) to a 96-well plate using a cold pipet tip. Air bubbles were carefully removed prior to gel polymerization and incubated at 37 °C for 1 h. In parallel, harvested pericytes and endothelial cells were pre-labeled with the cell tracker (Pericyte labeled with Green 5-chloromethylfluorescein diacetate (CMFDA; and endothelial cells labeled with Red CMTPX; CAS#: 942,416–35-5). Pericytes and endothelial cells were then mixed together (Pericyte at 4 × 10^5^ cells/ml and endothelial cells at 1.6 × 10^6^) and 200 µl of the mixed cell suspension was then added to each Matrigel™ coated well and incubated 16–18 h at 37 °C . Loss of pericyte coverage on the endothelial cells was monitored and imaged by microscope photographs using Olympus DP71 microscope.

## Result

### Morphine induces loss of pericytes and increases influx of monocytes *in vivo*

**FIGURE 1:**
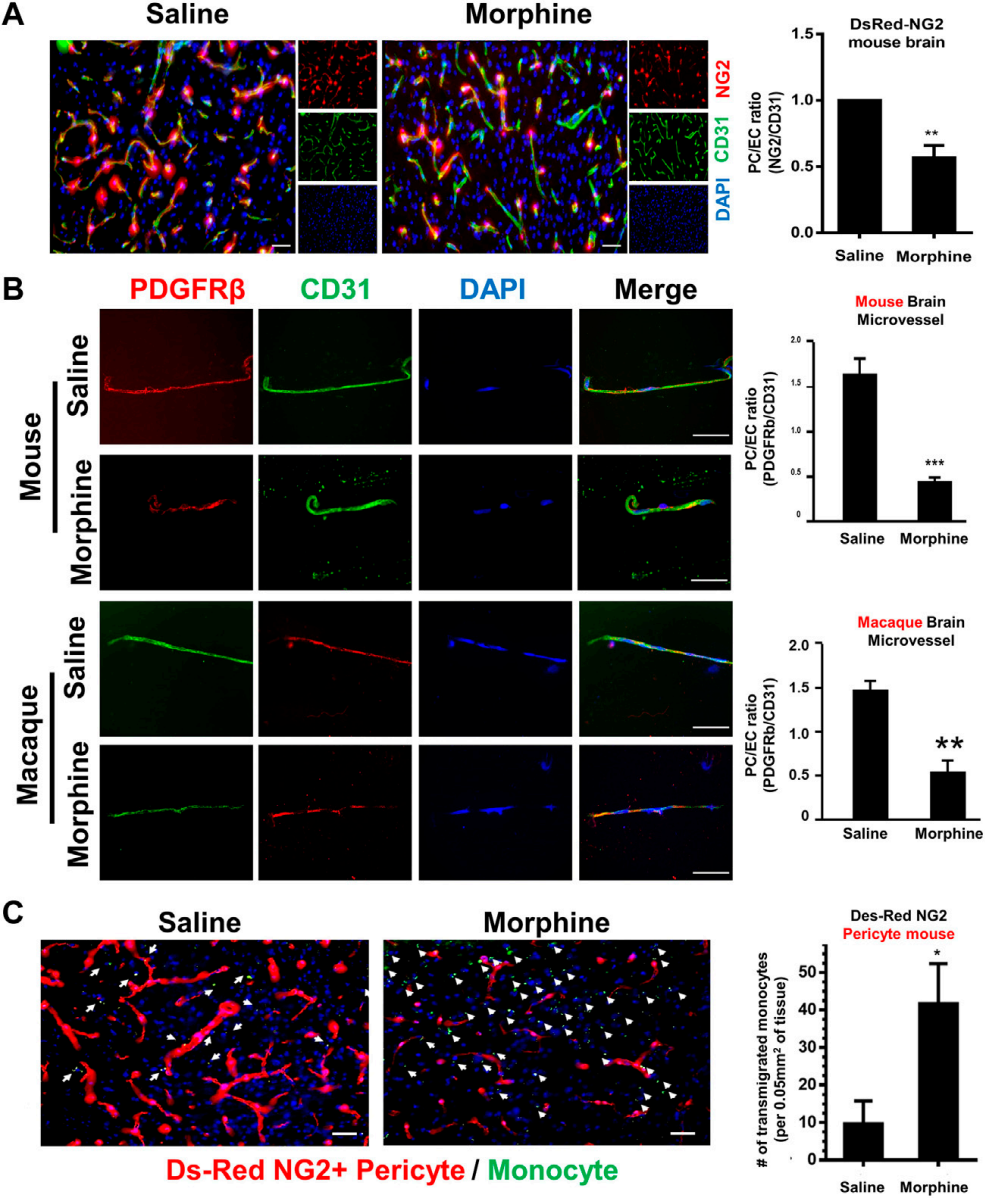
Morphine induces pericyte loss and influx of monocyte *in vivo*. **(A)** Representative immunostaining of endothelial cell marker CD31 (green) in brain sections of DsRed-NG2 mice-administrated with saline or morphine. *n* = 4 per group; bar, 20 μm; Quantification of ratio of NG2+ (Pericytes, red) fluorescent intensities area/CD31^+^ (Endothelial cells, green) fluorescent intensities area.Two-tailed Student’s t-test. **(B)** Representative images of double immunostaining of pericyte marker PDGFR-β (Green) and endothelial cell marker CD31 (Red) in microvessels isolated from either C57BL/6N mice or Macaque administrated with saline or morphine. Quantification of the ratio of PDGFR-β+ fluorescent intensities area/CD31^+^ fluorescent intensities area. **(C)** Representative images of CMFDA-labeled monocytes and DsRed-NG2 pericytes in the thalamus of mice administrated saline or morphine. *n* = 4 per group. Arrow: CMFDA-labeled monocyte (green color); pericytes (red color); bar, 20 μm; Quantification of CMFDA + cells in the thalamus. All data are presented as mean ± SD,*p < 0.05,**p < 0.01, ***p < 0.001 vs. saline group using Student’s t-test.

Transgenic mice expressing fluorescent pericytes (DesRed-NG2: DsRed under control of the proteoglycan NG2 promoter) (male, *n* = 4) were administrated either saline or morphine (intraperitoneally, an initial dose: 10 mg/kg; thrice a day) followed by ramping the dose by 5 mg/kg/day for six additional days (Cai et al., 2016). DesRed-NG2 mice administered morphine for 7 days, were sacrificed within 1 hour of the last morphine injection, and brains isolated. Brain sections were stained with anti-CD31 (Endothelial cells marker) antibody, followed by assessing pericyte coverage around the endothelium. As shown in Figure 1A, there was reduced ratio of NG2+ pericyte/CD31+ endothelial cells, indicating reduced pericyte coverage in morphine-administrated mice compared with the saline controls. To further confirm the phenomenon of morphine-induced loss of pericyte, microvessels isolated from C57BL/6N mice (male, *n* = 4) were administrated either saline or morphine for 7 days and were subjected to double immunostaining using antibodies specific for PDGFR-β (pericyte marker, green color) and CD31 (endothelial marker, red color). As shown in Figure 1B, there was reduced expression of the pericyte marker PDGFR-β in microvessels isolated from morphine-administrated mice. Additionally, increased pericyte loss was further recapitulated in microvessels isolated from the brains of morphine-administrated macaques, thereby confirming that morphine exposure induces loss of pericyte coverage both *ex vivo* and *in vivo*. Next, we sought to examine whether pericyte loss at the endothelium could result in enhanced monocyte infiltration. For this, DesRed-NG2 pericyte mice (described above), were injected with mouse bone marrow-derived monocytes (BMMs) on day 6, isolated from C57BL/6N mice, and pre-labeled with Green Cell Tracker CMFDA. The CMFDA-labeled BMMs were tail vein injected into the DesRed-NG2 pericyte mice. On day 7, at the end of morphine treatment, DesRed-NG2 mice were sacrificed within 1 h of morphine treatment and brains assessed for the distribution of CMFDA + monocytes. As shown in Figure 1C, there was significant increase in the numbers of CMFDA + monocytes that had transmigrated into the thalamus of mice administrated with morphine compared with the saline group. Additionally, the brains of morphine administered mice showed lesser NG2+ positive pericytes compared with the saline group. Taken together these findings indicated a negative correlation between pericyte coverage and monocyte influx, in the context of morphine administration.

### Morphine-ADEVs induce pericyte migration *in vitro*

**FIGURE 2:**
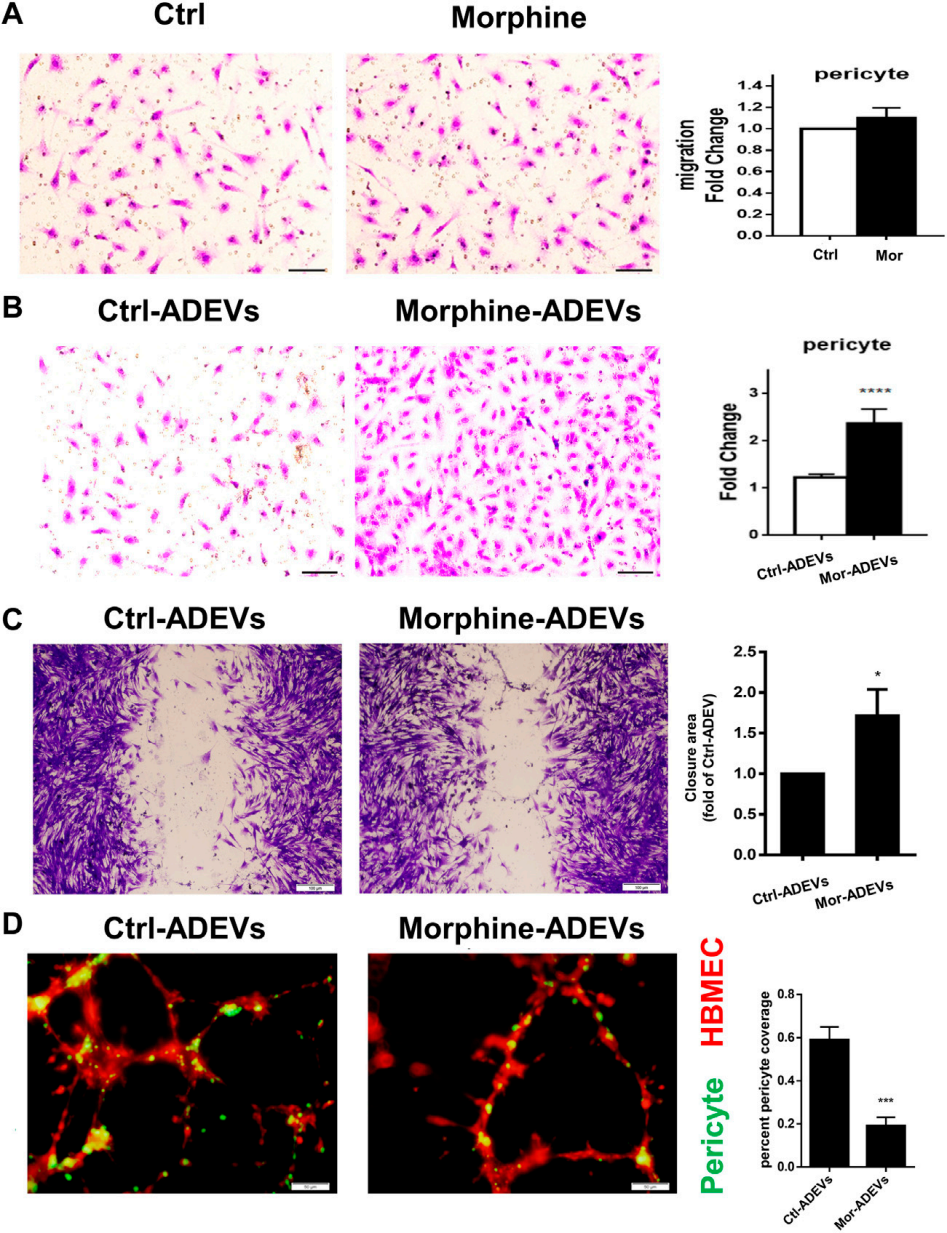
Morphine-ADEVs induce pericyte migration and loss *in vitro*. **(A)** The migration of pericytes that responded to morphine was measured by transwell migration assay. Migrated pericytes were stained with crystal violet. Scale bar = 20 µm. Quantification of migrated pericytes is shown on the right. N = 9 **(B)**The migration of pericytes responded to morphine-ADEVs was measured by transwell migration assay. Migrated pericytes were stained with crystal violet. Scale bar = 20 µm. Quantification of migrated pericytes is shown on the right. N = 9 **(C)** Migration of pericyte responded to morphine-ADEVs was measured by the scratch assay. The representative images of pericyte stained by crystal violet and then analyzed by ImageJ software. Scale bars, 100 μm. N = 9 **(D)** Loss of pericyte was measured in the 3D pericyte (green)-endothelia (Red) coculture model. The coverage of pericytes was analyzed by ImageJ. Scale bars, 50 μm. N = 9 All data are presented as mean ± SD of 3 individual experiments, *p < 0.05,**p < 0.01, ***p < 0.001 vs. control group using one-way ANOVA analysis.

Having demonstrated morphine-mediated pericyte loss *in vivo*, we next sought to examine the underlying mechanisms. Previous studies have demonstrated that pericyte migration away from the microvessels is one of the mechanisms involved in pericyte loss in various brain diseases (Pfister et al., 2008; Niu et al., 2014). For this, we first tested the direct effect of morphine on the pericyte migration *in vitro*. Based on an elegant review (Schulz et al., 2012) that summarizes the previous studies, the toxic morphine blood concentration in human abuse was reported to be 0.04–5 mg/L (0.14–17.5 μM). Based on this for our study, we chose the physiologically relevant dose of 10 μM, which has also been used routinely by others (He et al., 2011; Qiu et al., 2015)and us (Hu et al., 2018; Liao et al., 2020). Human primary pericytes were exposed to morphine (10 μM) for 24 h, and assessed for pericyte migration using the transwell migration assay. Morphine exposure failed to directly induce pericyte migration (Figure 2A). Since ADEVs play a critical role in regulating CNS cells, we next sought to study whether miRNA cargo in ADEVs could regulate pericyte migration. Previous studies (Chaudhuri et al., 2018; Hu et al., 2018) have shown upregulation of miR-23a in ADEVs isolated from morphine-stimulated astrocytes. Additionally, multiple lines of evidence demonstrate a key role of miR-23a in regulating cell migration (Tian et al., 2015; Huang et al., 2018; Xing et al., 2019). Furthermore, increased abundance of miR-23a has also been reported primarily in astrocytes in the CNS (Smirnova et al., 2005; Wang et al., 2008; Gioia et al., 2014; Brites and Fernandes, 2015). Taken together, we thus hypothesized that ADEV-miR-23a could regulate pericyte migration. To test this hypothesis, pericytes were exposed to either control- or morphine-ADEVs for 24 h, followed by assessing pericyte migration using the transwell migration assay. As shown in Figure 2B, morphine-ADEVs resulted in significant induction of pericyte migration. This phenomenon is further validated using the scratch migration assay. As shown in Figure 2C, morphine-ADEVs significantly induced pericyte migration compared with control-ADEVs.

Having demonstrated that morphine-ADEVs induced pericyte migration, the next question was whether pericyte migration resulted in reduced pericyte coverage on the endothelial cells. To answer this, we developed a pericyte-endothelial cells co-culture model to assess the loss of pericytes *in vitro*. Pericytes were exposed to either control- or morphine-ADEVs for 24 h, followed by labeling pericytes with the cell tracker CMFDA (green) while also labeling human brain microvascular endothelial cells (HBMECs) with cell tracker CMTPX (red). The cell tracker labeled pericytes and HBMECs were mixed at the ratio of 1:4 (pericyte: HBMEC), seeded onto the matrix gel, and cocultured for 24 h. Pericyte coverage on the HBMECs was monitored by confocal microscopy. As shown in Figure 2D, HBMECs (red) forming tube-like structures had pericytes (green) attached to them. There was a significant decrease in pericyte coverage on HBMECs exposed to morphine-ADEVs exposed pericytes compared with those exposed to control-ADEVs. These data thus indicated that morphine-ADEVs could induce loss of pericyte coverage *in vitro*.

### Morphine-ADEV induced pericyte migration involves transport of miR-23a from astrocytes to pericytes

**FIGURE 3:**
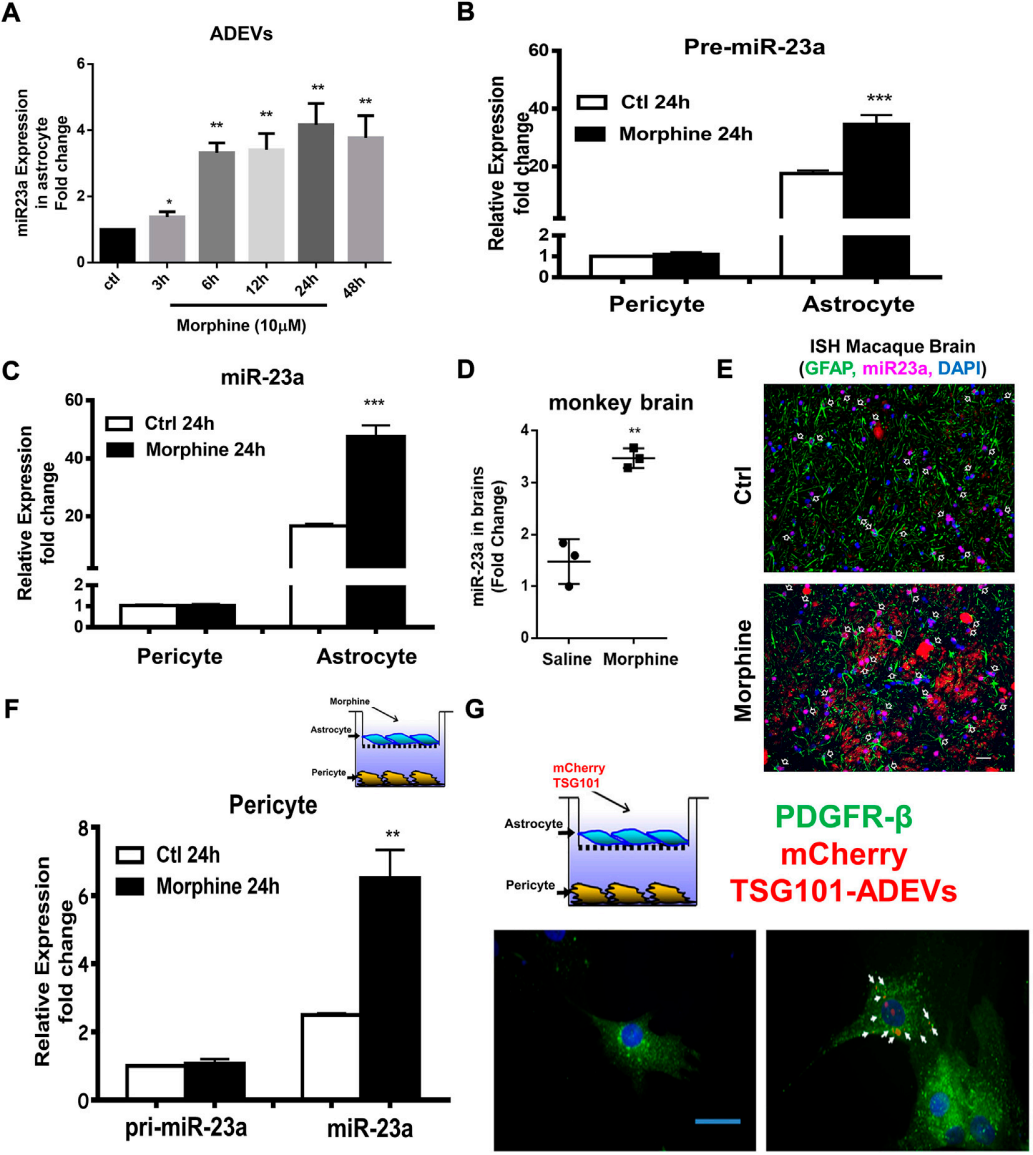
Morphine-ADVEs induce the upregulation of miR-23a in pericytes via ADEV-mediated transporting miR-23a from astrocytes to pericytes. **(A)** ADEVs isolated from astrocytes treated with or without morphine for indicated time points, followed by assessing miR-23a expression using real-time PCR. **(B,C)** Pericytes and astrocytes were treated with or without morphine for 24 h, followed by an assessment of pre- **(B)** or mature-miR-23a **(C)** expression using real-time PCR. **(D,E)** miR-23a was up-regulated in the brains of morphine-dependent macaques as assessed by **(D)** real-time PCR and, **(E)** *in situ* hybridization. **(F)** Exposure of astrocytes to morphine for 24 h, followed by coculturing with pericyte for an additional 24 h. Then extracted RNA from pericytes, followed by an assessment of pre- or mature-miR-23a in the pericytes. **(G)** mouse primary astrocytes were transfected with mCherry TSG101 plasmid for 24 h, followed by coculturing with pericytes for an additional 24 h. Paraformaldehyde fixed pericytes were permeabilized and stained for pericyte marker PDGFR-β (Green) and visualized by fluorescence microscopy. Scale bars, 20 μm. All data are presented as mean ± SD, *p < 0.05, **p < 0.01, ***p < 0.001 vs. control group using one-way ANOVA analysis.

To determine whether miR-23a plays a role in morphine-ADEV induced pericyte migration, we first examined the effect of morphine on the expression of miR-23a in the ADEVs. Human astrocytes were exposed to morphine for various time points (3–48 h) followed by assessing the expression of miR-23a in ADEVs isolated from conditioned media, of either control or morphine-exposed astrocytes, by real-time PCR. As shown in Figure 3A, morphine time-dependently induced the expression of miR-23a in ADEVs. Next, we validated morphine-mediated upregulation of miR-23a in the archival basal ganglia from morphine-dependent macaques. As shown in Figure 3D, there was increased expression of miR-23a in the basal ganglia of morphine-dependent macaques compared with the saline control group. Furthermore, these findings were also validated by *in situ* hybridization, wherein increased expression of miR-23a was observed in GFAP + astrocytes in the basal ganglia of morphine-dependent macaques (Figure 3E) compared with saline controls.

MiR-23a has been shown to be highly and exclusively expressed in astrocytes in the CNS (Smirnova et al., 2005; Wang et al., 2008; Gioia et al., 2014; Brites and Fernandes, 2015). We thus next sought to determine the expression levels of pre-miR-23a as well as mature miR-23a in both pericytes and astrocytes. As shown in Figures 3B,C, human astrocytes and pericytes were exposed to morphine for 24 h, followed by assessing the expression levels of pre-miR-23a (Figure 3B) and mature miR-23a (Figure 3C) by real-time PCR. Pericytes were shown to express pre- and mature-miR-23a at lower levels compared with the astrocytes. Exposure of pericytes to morphine did not alter miR-23a transcription (pre-miR-23a levels) and levels of mature miR-23a in pericytes. On the other hand, expression levels of both pre-miR-23a and mature-miR-23a were increased in morphine exposed astrocytes. These results thus indicated that astrocytes are the major source of elevated miR-23a in response to morphine stimulation. Next, we investigated whether miR-23a carried in morphine-ADEVs could be transferred from astrocytes to pericytes, leading, in turn, to migration of the latter, recipient cells. To this end, mCherry-TSG101 plasmid transfected astrocytes (to label ADEVs) were seeded on top of the transwell, and pericytes at the bottom of a 24-well plate. After 24 h of coculturing, cells were labeled for pericyte marker-PDGFR-β (Green) by immunostaining. As shown in Figure 3G, the signal of mCherry-TSG101-labeled ADEVs (red) from astrocytes was found in PDGFR-β+ pericytes, thus indicating uptake of ADEVs by pericytes. Next we determined whether miR-23a in morphine-ADEV could be taken up by the pericytes. Astrocytes were seeded in the top compartment of the transwell (on top) and exposed to morphine for 24 h, followed by coculturing with pericytes on the bottom of the transwell for an additional 24 h. Levels of pre- and mature-miR-23a in pericytes were assessed using real-time PCR. As shown in Figure 3F, mature-miR-23a but not pre-miR-23a was upregulated in pericytes cocultured with morphine-stimulated astrocytes in the coculture system, thus indicative of the fact that miR-23a in morphine-ADEVs was transferred from astrocytes into pericytes, thereby leading to upregulation of miR-23a in pericytes.

**FIGURE 4:**
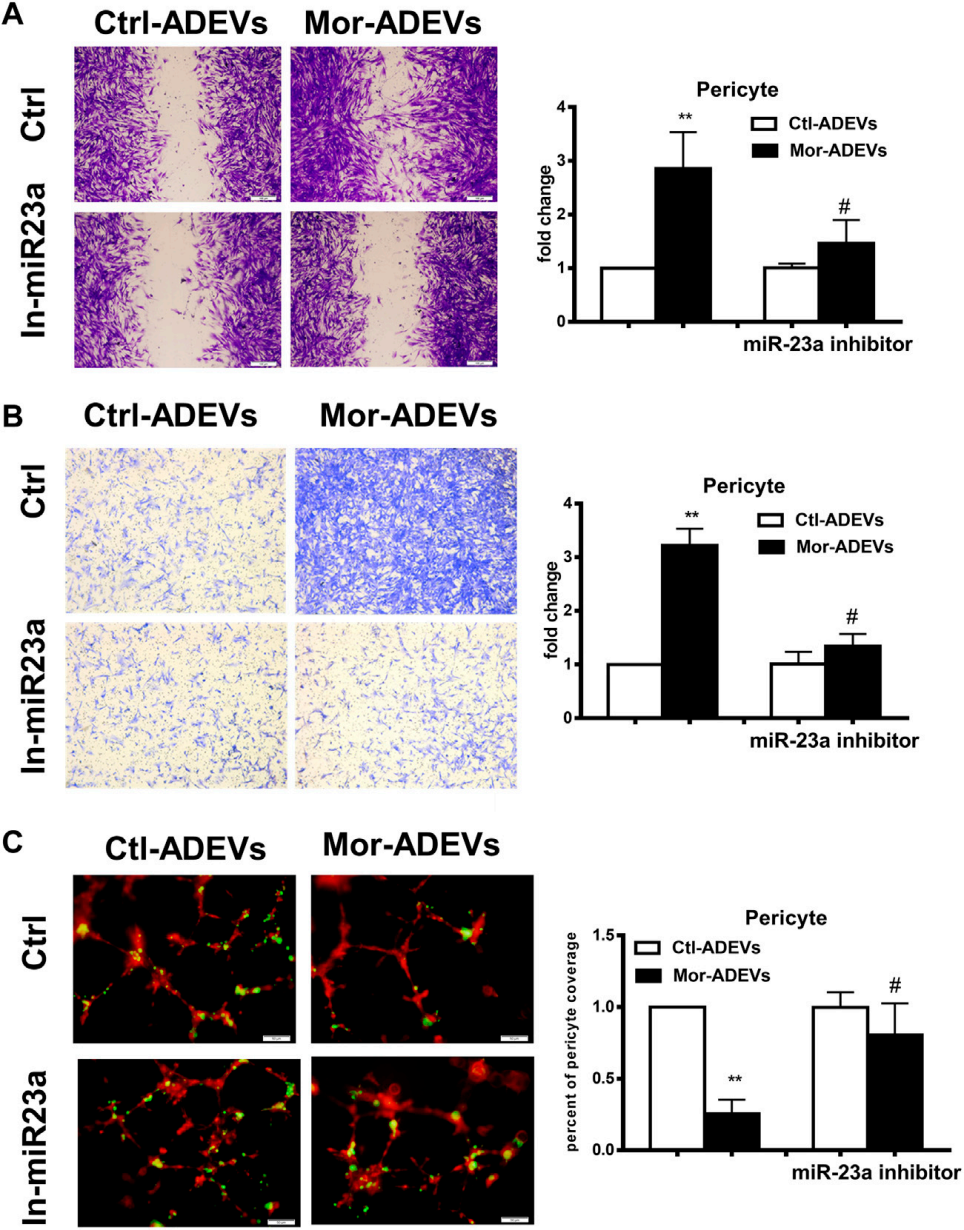
Morphine-ADVE-miR-23a induces pericyte migration. **(A)** The migration of pericytes was measured by the scratch migration assay. The representative images of pericytes were stained with crystal violet and then analyzed by ImageJ software. Scale bars, 100 μm. **(B)** The migration of pericytes was measured by the transwell migration assay. The representative images of migratory pericytes were stained by crystal violet and then analyzed by ImageJ software. Scale bar = 100 µm. **(C)** Loss of pericytes was measured by the 3D pericyte (green)-endothelia (Red) coculture model, and the representative images were analyzed by ImageJ. Scale bars, 50 μm. All data are presented as mean ± SD, **p < 0.01 vs. control group, #*p* < 0.05 vs. morphine-ADEVs + miR-23a inhibitor group using one-way ANOVA analysis.

We next sought to determine whether miR-23a was responsible for morphine-ADEV-induced pericyte migration. Pericytes were pre-transfected with the inhibitor of miR-23a for 24 h, followed by exposure of pericytes to either control- or morphine-ADEVs for an additional 24 h, and finally assessment of pericyte migration using the methods described above. As shown in Figure 4A, morphine-ADEVs significantly promoted pericyte migration in scratch assay, while morphine-ADEVs failed to induce migration in miR-23a inhibitor-transfected pericytes. This phenomenon was also validated using the transwell migration assay. ADEV-stimulated pericytes were seeded on the upper side of the transwell and migrating cells on the other side of the transwell were stained and quantified. As shown in Figure 4B, miR-23a inhibitor ameliorated morphine-ADEV-induced pericyte migration. Pericytes coverage was examined in the 3D pericyte-endothelial cells coculture model as well. As shown in Figure 4C, morphine-ADEVs significantly decreased the coverage of pericytes on endothelial cells, while morphine-ADEVs failed to cause pericyte loss in miR-23a inhibitor-transfected pericytes.

### HnRNP A2/B1-mediated sorting of miR-23a into ADEVs

**FIGURE 5:**
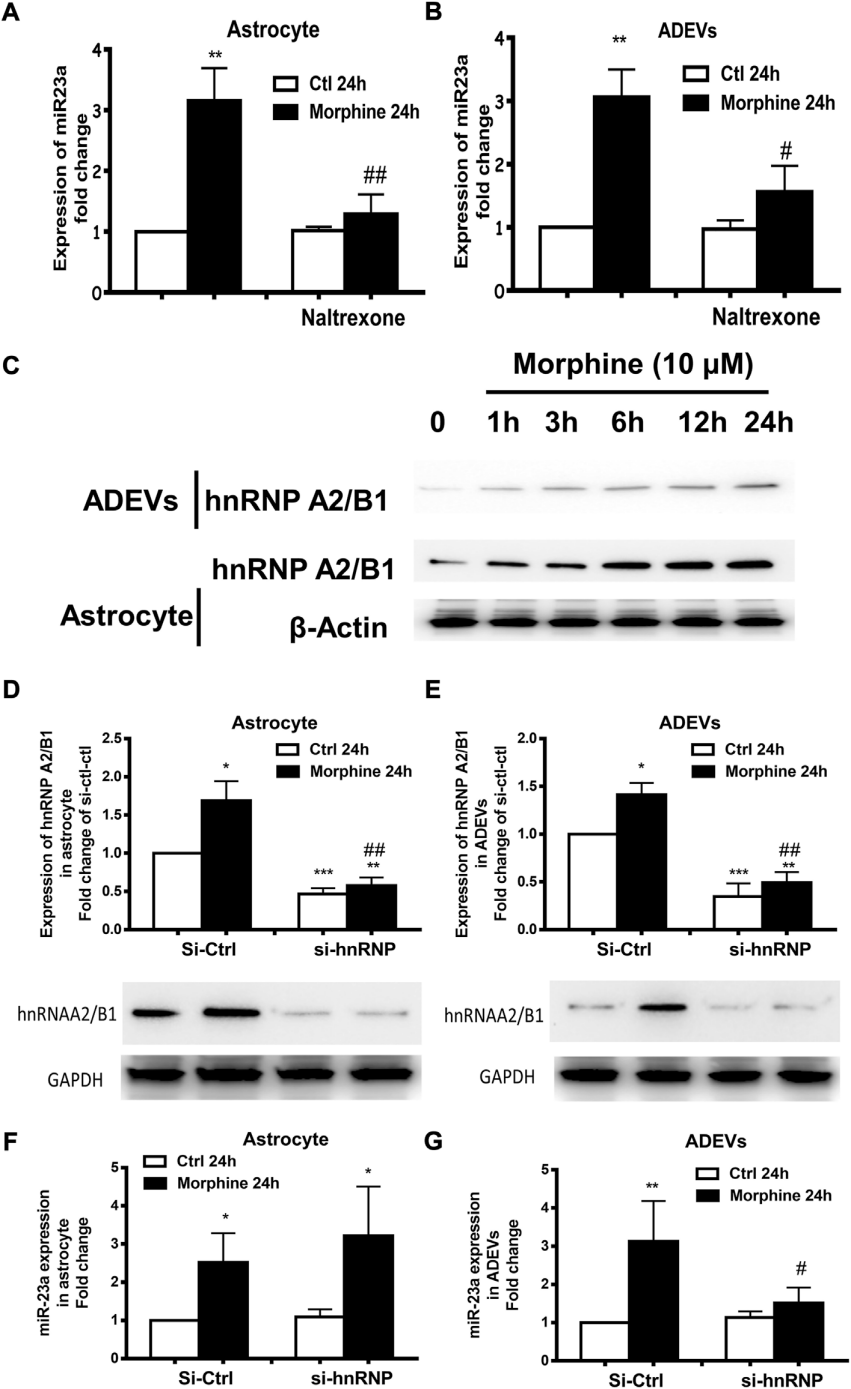
Morphine-mediated upregulation of miR-23a in ADEVs involves mu receptor and hnRNP A2/B1. **(A,B)** Astrocytes were pretreated with naltrexone (10 μM) for 1 h, followed by 24 h exposure to morphine. The expression of miR-23a in astrocytes **(A)** and ADEVs **(B)** was assessed using real-time PCR. Each set of results was quantified upon three independent experiments. **(C)** Representative western blot images of hnRNP A2/B1 expression in the lysates of cells and ADEVs from astrocytes exposed to morphine for various time points (1–24 h). **(D,E)** Representative western blot images of hnRNP A2/B1 expression in the lysates of cells **(D)** or ADEVs **(E)** from astrocytes transfected with control siRNA or si-hnRNP A2/B1 followed by morphine exposure. Each set of results was quantified upon three independent experiments. **(F,G)** Astrocytes were transfected with either control siRNA or si-hnRNP A2/B for 24 h, followed by morphine exposure for 24 h. The expression of miR-23a in astrocytes **(F)** and ADEVs **(G)** was assessed using real-time PCR. Each set of results was quantified upon three independent experiments. All data are presented as mean ± SD, *p < 0.05, **p < 0.01, ***p < 0.001 vs. si-ctl-control group; #*p* < 0.05, ##*p* < 0.01 vs. si-ctl-morphine group using one-way ANOVA analysis.

Morphine is a well-known mu receptor (one of the opioid receptors) agonist. The next logical step then was to examine whether morphine-mediated induction of miR-23a, either in astrocytes or ADEVs, involved the mu receptor. For this, astrocytes were pretreated with the opioid receptor antagonist naltrexone (10 μM) (Yue et al., 2008; Mohan et al., 2010) 1 h prior to morphine treatment, followed by extracting RNA from astrocytes as well as isolated ADEVs from the culture media. As shown in Figure 5A, pre-exposure of astrocytes with naltrexone significantly abrogated morphine-induced upregulation of miR-23a in both astrocytes and ADEVs (Figure 5B), thereby suggesting that morphine-mediated induction of miR-23a expression and release in astrocytes was mu opioid receptor-dependent.

Villarroya-Beltri et al. demonstrated a mechanism by which there is sumoylated heterogeneous nuclear ribonucleoprotein A2/B1 (hnRNP A2/B1)-mediated sorting of miRNAs into EVs (Villarroya-Beltri et al., 2013). This study identified specific motifs present in the miRNAs that could be the target of hnRNP A2/B1 for binding; its binding facilitates the sorting of these miRNAs into EVs. Intriguingly, we also found the hnRNP A2/B1 binding motif within the miR-23a sequence. We thus sought to explore whether morphine-mediated upregulation of miR-23a in morphine-ADEVs involved hnRNP A2/B1 complex. Astrocytes were first exposed to morphine for various time points (1–24 h), followed by assessing the expression levels of hnRNP A2/B1 proteins in the lysates of astrocytes and ADEVs by western blotting. We found a time-dependent increase of hnRNP A2/B1 proteins in morphine-stimulated astrocytes and in morphine-ADEVs (Figure 5C). Next, human primary astrocytes were transfected with either control siRNA or siRNA for hnRNP A2/B1 for 24 h, followed by exposure of cells to morphine for an additional 24 h, and then assessed for the expression of hnRNP A2/B1 proteins. As shown in Figure 5D, hnRNP A2/B1 siRNA transfection successfully knocked down the expression of hnRNP A2/B1 in astrocytes. Morphine failed to induce upregulation of hnRNP A2/B1 in astrocytes transfected with si-hnRNP A2/B1 compared with control siRNA transfected cells. Moreover, morphine failed to induce the upregulation of hnRNP A2/B1 in ADEVs isolated from hnRNP A2/B1 siRNA transfected astrocytes compared with control siRNA transfected cells (Figure 5E). In parallel, we examined the levels of miR-23a in these astrocytes and ADEVs and interestingly, found morphine-mediated upregulation of intracellular miR-23a in both hnRNP A2/B1 siRNA and control siRNA transfected astrocytes (Figure 5F). Morphine however, failed to upregulate miR-23a in the ADEVs isolated from astrocytes transfected with hnRNP A2/B1 siRNA (Figure 5G). These findings thus indicated that upregulation of miR-23a in morphine-ADEVs involved hnRNP A2/B1-mediated sorting of miR-23a into ADEVs.

### ADEV-miR-23a targets PTEN in pericytes

**FIGURE 6:**
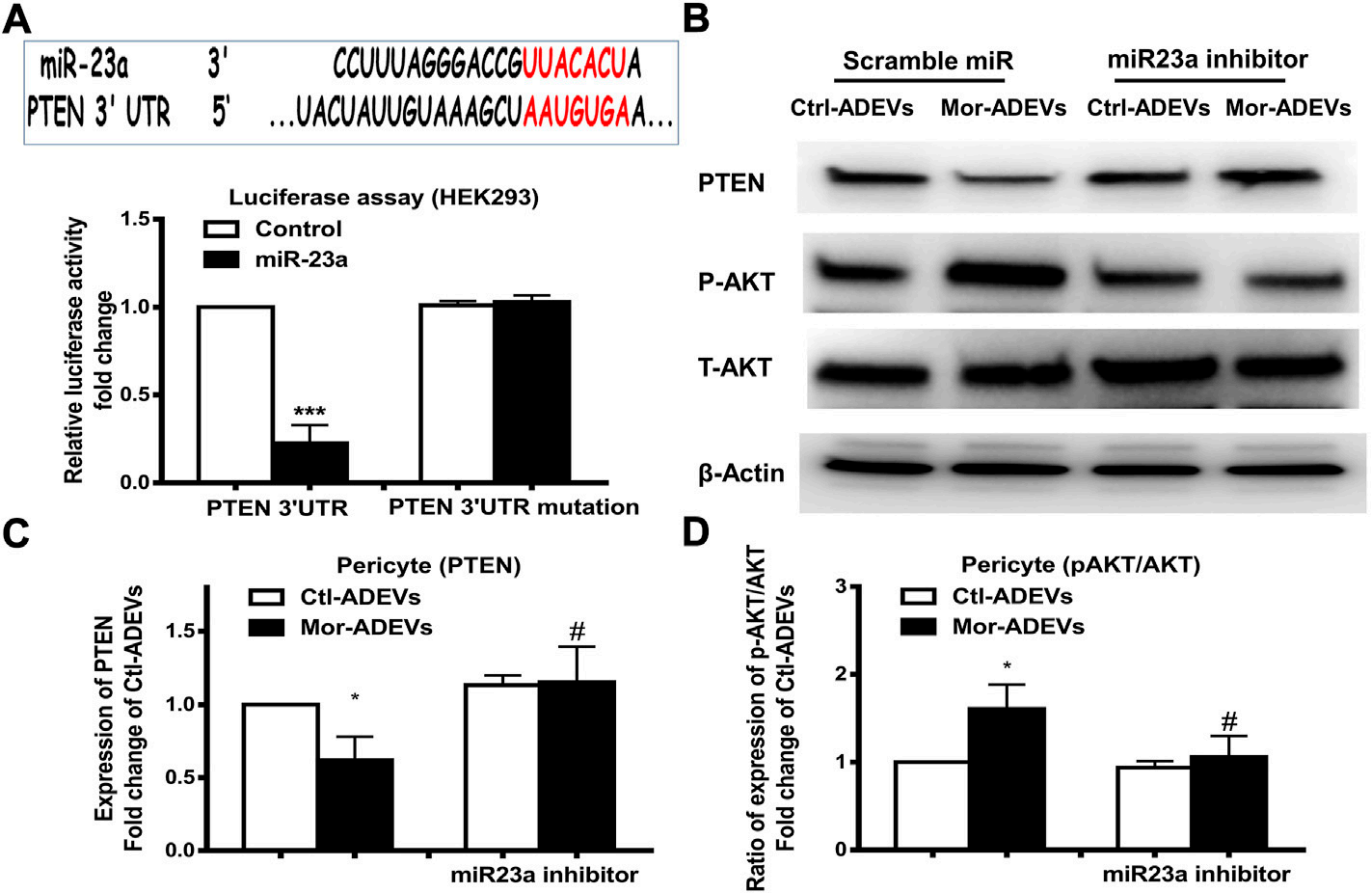
ADEVs-miR-23a targets PTEN that leads to activation of the AKT pathway. **(A)** Putative miR-23a binding site in PTEN mRNA. Relative luciferase activity of WT and 3′UTR mutant constructs of PTEN in HEK293 cells co-transfected with miR-control or miR-23a mimic. **(B)** Representative western blot and quantification of PTEN **(C)**, AKT and p-AKT **(D)** protein levels in pericytes transfected with either control-miR or miR-23a mimics for 24 h, followed by treatment of ctl-ADEVs or morphine-ADEVs. Each set of results was quantified upon three independent experiments. All data are presented as mean ± SD, *p < 0.05, ***p < 0.001 vs. ctl-ADVs group; #*p* < 0.05 vs. morphine-ADEVs group using one-way ANOVA analysis.

Having demonstrated morphine-induced upregulation of miR-23a in ADEVs, we next sought to examine whether ADEV-miR-23a could be taken up by pericytes and lead to pericyte migration. First, we explored the downstream target of miR-23a in pericytes. PTEN has been demonstrated as a target of miR-23a (Han et al., 2017; Wang et al., 2018). Additionally, it has been shown that PTEN negatively regulates cell migration by inhibiting the AKT pathway (Tian et al., 2015). We next sought to examine whether ADEV-miR-23a could bind directly to the 3′-untranslated region (3′UTR) of the PTEN mRNA, leading to translational inhibition by performing reporter luciferase assay. For this, HEK293 cells were co-transfected with miR-23a mimic and PTEN-3′UTR-luciferase constructs, or its mutation constructs, followed by the assessment of luciferase activity. As shown in Figure 6A, miR-23a mimic resulted in a significant decrease in luciferase activity, indicating thereby that PTEN was a direct target of miR-23a. In cells co-transfected with the PTEN 3′UTR mutant however, miR-23a mimic failed to reduce luciferase activity (Figure 6A). Further confirmation of the role of ADEV-miR-23a in regulating PTEN translation was carried out by transfecting pericytes with an inhibitor of miR-23a, followed by exposure of pericytes to either control-ADEV or morphine-ADEV and subsequently assessing the expression of the PTEN protein by western blot. As shown in Figures 6B,C and as expected, morphine-ADEVs significantly reduced the expression of the PTEN protein compared with cells exposed to control-ADEVs. In contrast, morphine-ADEVs failed to decrease the expression of PTEN in cells co-transfected with the miR-23a inhibitor. We next examined the phosphorylation of AKT in pericytes transfected with miR-23a inhibitor followed by exposure to cells to either control-ADEV or morphine-ADEV. As shown in Figures 6B,D, morphine-ADEVs activated AKT pathway by inducing AKT phosphorylation in pericytes. Morphine-ADEVs however, failed to activate the AKT pathway, in cells transfected with miR-23a inhibitor.

### Morphine-ADEV-mediated pericyte migration involves the miR-23a/PTEN/AKT axis

**FIGURE 7:**
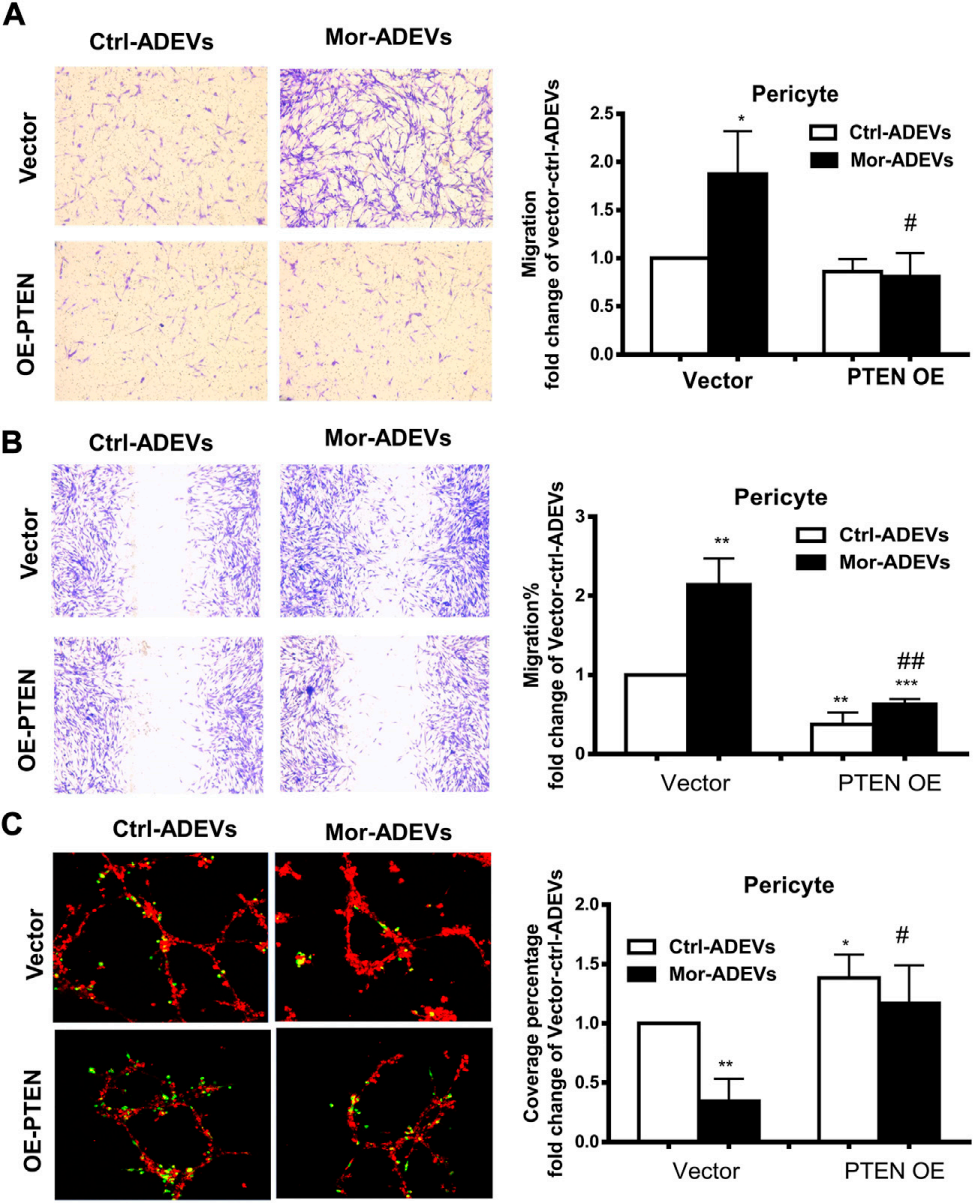
Overexpression of PTEN attenuates morphine-ADEV-mediated pericyte migration. **(A–C)** Pericytes were transfected with either control vector or PTEN overexpression plasmid (PTEN OE) for 24 h, then exposed to either control-ADEVs or morphine-ADEVs. Then the migration of pericyte was measured by transwell migration assay **(A)**. The representative images of migratory pericytes stained with crystal violet. Scale bar = 100 µm. The migration of pericyte was measured by the scratch assay **(B)**. The representative images of pericytes stained with crystal violet and then the representative images were analyzed by ImageJ software. Scale bars, 100 μm. Loss of pericyte was measured by 3D pericyte (green)-endothelia (Red) coculture model **(C)**, and then the representative images were analyzed by ImageJ. Scale bars, 50 μm. All data are presented as mean ± SD, *p < 0.05, ***p < 0.01 vs. control group (Vector-CTL-ADEVs), #*p* < 0.05, ##*p* < 0.01 vs. Vector-morphine-ADEVs group using one-way ANOVA analysis.

To further confirm whether PTEN was a key player in ADEV-miR-23a-mediated pericyte migration, pericytes were transfected with either PTEN overexpression construct or a control vector, followed by exposure of pericytes to control-ADEVs or morphine-ADEVs and assessed for pericyte migration using various methods described above. As shown in Figure 7A, morphine-ADEVs significantly promoted pericyte migration in control vector-transfected cells in the scratch assay, while morphine-ADEVs failed to induce pericyte migration in cells transfected with PTEN overexpressing plasmid. We validated this phenomenon using the transwell migration assay wherein ADEV-stimulated pericytes were seeded onto the upper side of the transwell, and migrated cells on the other side of transwell stained and quantified. As shown in Figure 7B, morphine-ADEVs induced migration of pericytes transfected with control vector, but not pericytes transfected with PTEN overexpressing constructs. Next, we examined pericyte coverage in the 3D pericyte-endothelial cells coculture model. As shown in Figure 7C, morphine-ADEVs significantly decreased pericyte coverage of endothelial cells, and morphine-ADEVs failed to cause pericyte loss in PTEN overexpressing cells.

**FIGURE 8:**
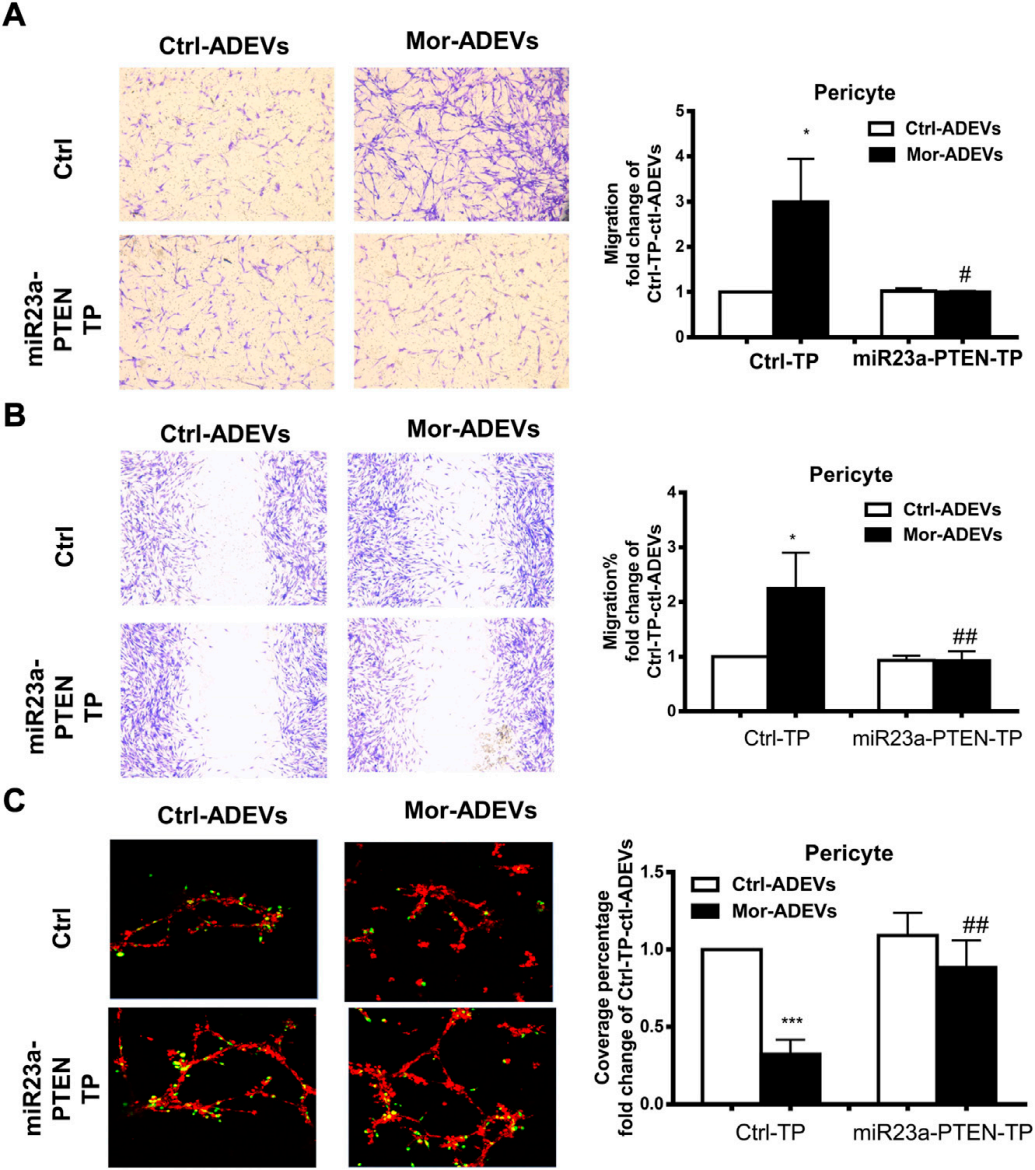
miR-23a-PTEN-TP attenuates morphine-ADEV-mediated pericyte migration. **(A–C)** Pericytes were transfected with either oligo of target protector control (CTL-TP) or miR-23a-PTEN-target protector (miR-23a-PTEN-TP) for 24 h, then exposed to either control-ADEVs or morphine-ADEVs. Then the migration of pericyte was measured by transwell migration assay **(A)**. The representative images of migratory pericytes stained with crystal violet. Scale bar = 100 µm. The migration of pericyte was measured by the scratch assay **(B)**. The representative images of pericytes stained with crystal violet and then images were analyzed by ImageJ software. Scale bars, 100 μm. Loss of pericyte was measured by 3D pericyte (green)-endothelia (Red) coculture model **(C)**, and then the representative images were analyzed by ImageJ. Scale bars, 50 μm. All data are presented as mean ± SD,*p < 0.05,**p < 0.01, ***p < 0.001 vs. control group (CTL-TP-CTL-ADEVs), #*p* < 0.05, ##*p* < 0.01 vs. CTL-TP-morphine-ADEVs group using one-way ANOVA analysis.

In keeping with these findings, we transfected pericytes with either miR-23a-PTEN target protector oligo (miR-23a-PTEN-TP) that specifically protects the miR-23a binding site on the endogenous PTEN 3′UTR or a scrambled target protector oligo, followed by exposure of pericytes to either control-ADEVs or morphine-ADEVs and assessed for pericyte migration. As shown in Figure 8A, morphine-ADEVs significantly promoted the migration of pericytes transfected with scrambled target protector oligo in the scratch assay, while morphine-ADEVs failed to induce migration of pericytes transfected with miR-23a-PTEN-TP. These findings were validated using the transwell migration assay. As shown in Figure 8B, morphine-ADEVs induced migration of pericytes transfected with scrambled target protector oligo, but not of pericytes transfected with miR-23a-PTEN-TP oligo. Next, we examined the pericyte coverage in the 3D pericyte-endothelial cell coculture model following miR-23a-PTEN-TP oligo transfection and stimulation with morphine-ADEVs. As shown in Figure 8C, morphine-ADEVs significantly down regulated pericyte coverage in cells transfected with scrambled target protector oligo. Morphine-ADEVs failed to cause pericytes loss in miR-23a-PTEN-TP oligo transfected pericytes.

## Discussion

It is well known that morphine abuse and HIV infection are closely linked (Wang and Ho, 2011; Reynolds et al., 2012; El-Hage et al., 2015; Vaidya et al., 2016). Many studies have demonstrated that morphine not only potentiates the progression of HIV infection but can also exacerbate neurological complications associated with the disease (Murphy et al., 2019). Morphine has a detrimental effect on most CNS cells, such as the endothelium (Lam et al., 2007), astrocytes (Stiene-Martin et al., 1991; Sil et al., 2018), microglia (Bokhari et al., 2009; Ryu et al., 2018) and neurons (Smith et al., 2012), thus contributing to the pathogenesis of HIV-associated neurocognitive disorders (HAND). In the current study, we demonstrate a novel molecular mechanism underlying morphine-mediated induction of miR-23a in ADEVs, that are taken up by the pericytes resulting in downregulation of PTEN, which, in turn, leads to pericyte migration. This leads to enhanced influx of monocytes in the CNS, ultimately contributing to neuroinflammation.

Pericytes have been considered key players in maintaining the integrity of the BBB. Several studies have demonstrated that pericyte coverage is positively correlated with the integrity of the BBB (Bell et al., 2010; Proebstl et al., 2012; Stark et al., 2013). Increased loss of pericyte coverage on the vessels correlated with increased influx of immune cells in the brain. Drugs of abuse such as opioids are well-recognized to cause BBB breach, which, in turn, leads to increased influx of monocytes and ensuing neuroinflammation. The role of pericytes in morphine-mediated neuroinflammation, however, remains less understood. Herein, our *in vivo* studies demonstrate morphine-mediated loss of pericytes in the brain microvessels, which results in influx of inflammatory monocytes in the brain. Furthermore, these findings were also validated *ex vivo*, wherein there were reduced numbers of PDGFR-β+ pericytes in microvessels isolated from morphine-administrated mice and macaques. Interestingly, the migration of pericytes away from the vessel wall has been demonstrated in several electron microscopic studies, in the context of ischemia and reperfusion disease models (Takahashi et al., 1997; Gonul et al., 2002; Melgar et al., 2005). The functional outcome of pericyte migration and where they move to, however, remains unknown. Intriguingly, detachment of pericytes from the microvessels was also demonstrated in a model of traumatic brain injury (TBI) (Dore-Duffy et al., 2000). In this study it was found that the remaining pericytes on the vessel showed cytoplasmic changes consistent with degeneration. These findings indicated two outcomes: 1) That pericyte migration could be a mechanism evolved by pericytes to bypass cell death in the context of TBI; 2) Non-migrating pericytes could die rapidly resulting in pericyte loss. Based on our findings in the current study, we speculate that loss of pericyte coverage from the endothelial cells is not likely a result of pericyte death. We exposed pericytes to either morphine or morphine-ADEVs and assessed cell viability using MTT assay. Morphine or morphine-ADEVs failed to induce pericyte death (data not shown). The question of where the pericytes migrate remains. Future studies using two-photon microscopy to track pericyte migration real-time *in vivo* could help delineate the site of pericyte migration within the brain.

Additionally, since pericytes have been shown to exhibit phagocytotic activity both *in vitro* (Pieper et al., 2014) and *in vivo* (Ma et al., 2018), it is plausible to also speculate that the pericytes could likely migrate to the damaged CNS cells (neurons, astrocytes, or microglial cells) in an effort to clear the cellular debris. Furthermore, owing to the multipotent nature of pericytes (Dore-Duffy et al., 2006) and their ability to differentiate, it is also possible that these cells could be primed to replace dying cells by differentiating into neuronal (Karow et al., 2018), astroglial (Dore-Duffy et al., 2006), microglial (Sakuma et al., 2016), or oligodendrocytic (Kim et al., 2021) phenotypes. This could be another possibility of pericyte fate after migration and needs further investigation.

Emerging evidence demonstrates miRNAs are key regulators in almost every cellular process. Generally, miRNAs control gene expression at the post-transcription level by regulating degradation or translational repression of target mRNAs (Catalanotto et al., 2016). Our current study demonstrated that morphine-mediated dysregulation of miRNA cargo in ADEVs leads to pericyte migration and loss of pericyte coverage with accompanying increased influx of monocyte-associated neuroinflammation in the brain. We demonstrated that exposure of human primary astrocytes to morphine resulted in upregulation and release of miR-23a in ADEVs involving a mu-receptor-dependent manner. This is in agreement with previous studies (Hwang et al., 2012; Toyama et al., 2017), demonstrating miR-23a is an opioid-receptor-regulated miRNA.

Furthermore, we also demonstrate morphine-mediated upregulation of ADEV-miR-23a involves a hnRNP A2/B1-mediated sorting mechanism. The RNA binding protein hnRNP A2/B1 has been reported to play a role in sorting miRNAs into EVs. For example, Villarroya-Beltri *et. al.* demonstrated sumoylated hnRNP A2/B1-mediated sorting of miRNAs into EVs *via* binding to the exosomal-sorting motif (such as GGAG, UGAG, GGCC, and GCCA) (Villarroya-Beltri et al., 2013). Interestingly, there is a hnRNP A2/B1 binding motif within miR-23a (GCCA). Exposure of human astrocytes to morphine resulted in a time-dependent upregulation of hnRNP A2/B2 in both astrocytes and ADEVs. We showed that knockdown of hnRNP A2/B1 attenuated both morphine-mediated upregulation of miR-23a in ADEVs as well as pericyte migration. It is likely that hnRNP A2/B1 could potentially contribute to various cellular dysfunctions such as pericyte migration and loss *via* the downstream miRNAs. Moreover, hnRNP A2/B1 could be envisioned as a novel target for intervention strategies aimed at blocking morphine-mediated pericyte loss and associated neuroinflammation.

PTEN, a tumor suppressor gene, has been well-studied in the field of cancer (Tamura et al., 1999). Loss or mutation of PTEN has been observed in ∼45% of endometrial cancers, ∼30% of glioblastomas (Tamura et al., 1999). In cancers of prostate, glioblastoma, lung, and breast cancer, PTEN is expressed at lower levels, (DeGraffenried et al., 2004; Zhu et al., 2013). Depletion of PTEN results in phosphorylation of FAK (Tamura et al., 1999) and activation of the Akt/PKB pathway (Chin and Toker, 2009), in turn, leading to the promotion of cell invasion, migration, and growth. Intriguingly, it has been shown by Zhang et al. (2015) that astrocyte-derived exosomal miR-19a-mediated loss of PTEN expression in cancer cells promoted brain metastasis. Additionally, our previous study also demonstrated that astrocyte-derived EV-miR-9-mediated downregulation of PTEN promoted microglial migration in the context of HIV Tat stimulation (Yang et al., 2018). In the current study, we show that exposure of astrocytes to morphine resulted in upregulation of ADEV-miR-23a, that could be taken up by pericytes, targeting PTEN, in turn, leading to pericyte migration. We also show that blocking miR-23a and overexpressing PTEN resulted in attenuation of morphine-ADEV-mediated pericyte migration. These findings expand our understanding of the role of PTEN in morphine-mediated pericyte loss and neuroinflammation.

In summary, our findings demonstrate a novel mechanism by which morphine-mediated pericyte migration resulted in pericyte loss and influx of monocytes *in vivo*, involving the release of ADEV-miR-23a and uptake of ADEVs by pericytes with activation of the PTEN/Akt pathway. These findings have implications for opioid abusers who have increased risks of neuroinflammation and cognitive decline.

## Data Availability

The raw data supporting the conclusion of this article will be made available by the authors, without undue reservation.
